# Giant enhancement of optoelectronic properties in compressed boron-rich semiconductors

**DOI:** 10.1093/nsr/nwag051

**Published:** 2026-01-27

**Authors:** Ming-Xing Huang, Kun Ye, Jingyu Hou, Yufei Gao, Guochun Yang, Lin Wang, Wentao Hu, Bo Xu, Zhongyuan Liu, Xiao-Ji Weng, Feng Ke, Xiang-Feng Zhou, Yongjun Tian

**Affiliations:** Center for High Pressure Science, State Key Laboratory of Metastable Materials Science and Technology, Yanshan University, Qinhuangdao 066004, China; Hebei Key Laboratory of Microstructural Material Physics, School of Science, Yanshan University, Qinhuangdao 066004, China; School of Electronics and Information Engineering, Institute of Quantum Materials and Devices, State Key Laboratory of Separation Membrane and Membrane Processes, Tiangong University, Tianjin 300387, China; Center for High Pressure Science, State Key Laboratory of Metastable Materials Science and Technology, Yanshan University, Qinhuangdao 066004, China; Center for High Pressure Science, State Key Laboratory of Metastable Materials Science and Technology, Yanshan University, Qinhuangdao 066004, China; Hebei Key Laboratory of Microstructural Material Physics, School of Science, Yanshan University, Qinhuangdao 066004, China; Center for High Pressure Science, State Key Laboratory of Metastable Materials Science and Technology, Yanshan University, Qinhuangdao 066004, China; Center for High Pressure Science, State Key Laboratory of Metastable Materials Science and Technology, Yanshan University, Qinhuangdao 066004, China; Center for High Pressure Science, State Key Laboratory of Metastable Materials Science and Technology, Yanshan University, Qinhuangdao 066004, China; Center for High Pressure Science, State Key Laboratory of Metastable Materials Science and Technology, Yanshan University, Qinhuangdao 066004, China; Center for High Pressure Science, State Key Laboratory of Metastable Materials Science and Technology, Yanshan University, Qinhuangdao 066004, China; Center for High Pressure Science, State Key Laboratory of Metastable Materials Science and Technology, Yanshan University, Qinhuangdao 066004, China; Center for High Pressure Science, State Key Laboratory of Metastable Materials Science and Technology, Yanshan University, Qinhuangdao 066004, China; Hebei Key Laboratory of Microstructural Material Physics, School of Science, Yanshan University, Qinhuangdao 066004, China; Center for High Pressure Science, State Key Laboratory of Metastable Materials Science and Technology, Yanshan University, Qinhuangdao 066004, China

**Keywords:** borides, optoelectronic, high pressure, anti-Wilson

## Abstract

Optoelectronic devices often experience performance degradation under extreme conditions, such as elevated dark current and reduced sensitivity. Here we demonstrate that pressure uniquely enhances the optoelectronic performance of boron-rich semiconductor AlCu_1-δ_B_25_ via an unconventional anti-Wilson effect. Under compression, the photocurrent of AlCu_1-δ_B_25_ increases by more than 20-fold, to ∼7.22 μA at 26.5 GPa, while the dark current reduces dramatically by nearly four orders of magnitude (to ∼0.2 nA), yielding an unprecedented improvement in the on/off ratio exceeding 10^5^-fold. Simultaneously, pressure significantly accelerates the optoelectronic response, reducing the response time by three orders of magnitude. Optical absorption measurements reveal an anomalous pressure-driven anti-Wilson effect in AlCu_1-δ_B_25_. First-principles calculations indicate that this anomaly arises from an upward shift of Al-3*s* states through interactions with B-2*s* electrons. Our findings underscore the significance of the anti-Wilson effect in optimizing optoelectronic properties and establish boron-rich semiconductors as promising candidates for harsh-environment devices.

## Teaser

Pressure-induced bandgap widening in boron-rich semiconductors AlCu_1-δ_B_25_ synergistically optimizes the on/off ratio and response time, offering a novel strategy for enhancing optoelectronic performance.

## INTRODUCTION

Over decades, significant progress has been made in the development of diverse optoelectronic materials, which have found broad applications in photodetectors, photovoltaics, and light-emitting diodes [1–4]. A fundamental challenge for optoelectronic devices is achieving reliable performance in extreme environments, such as high temperature, pressure, and mechanical stress. Conventional semiconductors often experience rapid degradation of their critical physical properties and mechanical robustness under these harsh environments, resulting in severe performance deterioration including diminished on/off ratio, elevated dark current, prolonged response time, and other functional impairments. Recently, extensive studies have focused not only on evaluating the performance reliability of optoelectronic semiconductors under pressure, but also on actively optimizing their functionality via pressure or stress [5–16]. Notably, pronounced photocurrent enhancement has been achieved via pressure engineering, offering an effective route toward device performance optimization. However, pressure simultaneously introduces performance trade-offs. Pressure generally enhances orbital hybridization, narrows bandgaps, and even drives an insulator-to-metal transition (known as the Wilson transition), which inevitably elevates dark current, impairs switching speed [12–16], and degrades the overall performance of optoelectronic devices. To overcome these drawbacks, optoelectronic materials exhibiting an anomalous anti-Wilson effect, i.e. bandgap widening under pressure, are highly desirable, as this effect suppresses dark current, a key benefit that minimizes background noise and reduces power consumption during switching. Furthermore, materials with high mechanical strength and stiffness are also essential in order to resist deformation and performance degradation under pressure.

Recently, boron-rich semiconductors have emerged as a compelling functional material class [17–29]. Their robust B-B covalent frameworks enable extraordinary physical properties including high stiffness, exceptional hardness (>25 GPa), and ultrahigh thermal stability (>2000°C) [30–33]. The strong covalent bonding supports broadband absorption and luminescence, as exemplified by the pronounced photoconductivity in boron carbide under X-ray/UV irradiation [34,35]. In recent studies, Zheng *et al.* developed a *p*-type semiconductor Al_2.69_B_50_, which exhibits excellent optical transmittance in the visible range and ultrahigh hardness exceeding 40 GPa [36]. Meanwhile, Huang *et al.* improved the conductivity of a boron-rich material, Cu_2−δ_B_25_, by creating conductive channels between B_12_ icosahedral building blocks [37]. These advances highlight the potential of boron-rich materials for electronic devices operating under extreme high-temperature/pressure conditions. Motivated by these findings, we aim to investigate the optoelectronic properties of boron-rich semiconductors under pressure, and further explore the role of the pressure-driven anti-Wilson effect in enhancing carrier dynamics and optoelectronic performance.

In this study, we demonstrate that, deviating from typical pressure-induced performance degradation and trade-offs, pressure substantially enhances the optoelectronic performance of boron-rich semiconductor AlCu_1-δ_B_25_ via an anti-Wilson effect. The AlCu_1-δ_B_25_ photocurrent increases by more than 20-fold up to 26.5 GPa, while the dark current reduces dramatically by nearly four orders of magnitude (to ∼0.2 nA), yielding an unprecedented improvement in the on/off ratio exceeding 10^5^-fold. Simultaneously, the response time shows a dramatic reduction by three orders of magnitude. Optical absorption measurements and density-functional-theory (DFT) calculations reveal an anomalous anti-Wilson effect caused by interactions between Al-3*s* and B-2*s* electrons.

## RESULTS AND DISCUSSION

**Figure 1.**
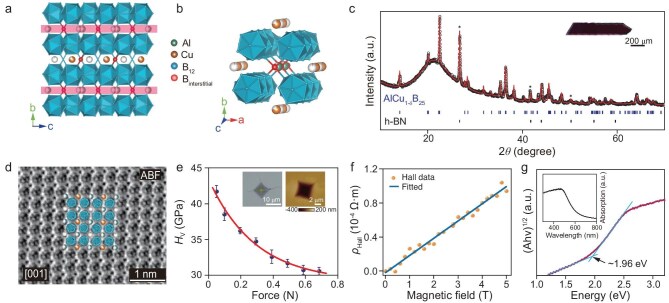
Ambient-pressure characterizations of AlCu_1-δ_B_25_ synthesized via high-temperature and high-pressure method. (a and b) Crystal structure viewed along the [100] (a) and [001] (b) orientations. (c) Le Bail fitting of the XRD pattern using the tetragonal *P*-4*n*2 structure (open circles: experimental data; red line: fitting results, black asterisks: h-BN peaks from the residual crucible material used for high-temperature and high-pressure synthesis). Inset: photograph of the synthesized single-crystal sample. (d) Annular-bright-field images along the [001] direction. (e) Vickers hardness test (*H*_V_) results of the synthesized single crystal. Inset: optical and atomic force microscopy images of the indentation area. (f) Hall data collected at 300 K. (g) Tauc plot of the UV-visible absorption spectrum collected at ambient pressure.

Single-crystal Al-Cu-B compounds were synthesized via a high-temperature, high-pressure method. Ambient-pressure characterization results are shown in Fig. 1 and Figs S1–S4. Energy dispersive X-ray spectroscopy indicates that the average atomic ratios of the synthesized single-crystal is Al:Cu:B ≈ 1.01:0.67:25 (Fig. S1). Although minor variations in the atomic ratios are observed across different samples and measured positions, the material’s physical properties remain insensitive to such compositional fluctuations. For simplicity, we refer to the synthesized compounds as AlCu_1-δ_B_25_ (δ = 0.3∼0.4). X-ray diffraction (XRD) indicates that the crystalline structure of AlCu_1-δ_B_25_ is consistent with a tetragonal structure (*P*-4*n*2 symmetry) with lattice constants *a* = *b* = 9.002 ± 0.003 Å and *c* = 5.069 ± 0.002 Å (Fig. 1a–c), and is comparable to previously reported results [38]. This structure is constructed by insertion of Al and Cu atoms into an α-tetragonal boron framework (including four B_12_ icosahedra and two interstitial B atoms per unit cell), where Al atoms occupy the 2a Wyckoff positions and Cu atoms partially occupy the 2c/2d sites. Aberration-corrected scanning transmission electron microscopy confirms the partial occupancies of Cu atoms and compositional fluctuations, as evidenced by spatially varied peak intensities at their respective lattice sites (Fig. 1d and Fig. S2).

The Vickers hardness test shows that AlCu_1-δ_B_25_ exhibits a hardness of ∼30.4 GPa at ambient conditions (Fig. 1e), suggesting its excellent mechanical properties. Thermogravimetric analysis and differential scanning calorimetry show no identifiable mass loss and endothermic/exothermic process with temperatures up to 1400°C, confirming the thermal stability of this boron-rich material (Fig. S3). Resistivity and Hall effect measurements (Fig. 1f and Fig. S4) identify AlCu_1-δ_B_25_ as a *p*-type semiconductor, with a room-temperature conductivity of ∼0.433 S/cm, a carrier concentration of ∼1.03 × 10^16^ cm^−3^, and a high carrier mobility of ∼262.1 cm^2^V^−1^s^−1^. The hole carrier mobility is comparable to that of two-dimensional transition metal chalcogenides [39,40]. Furthermore, ambient-pressure absorption experiments indicate that AlCu_1-δ_B_25_ has an indirect bandgap of ∼1.96 eV (Fig. 1g), locating in the visible light spectrum range. The combination of semiconducting behavior, high carrier mobility, sizable optical bandgap, superior hardness, and thermal stability make AlCu_1-δ_B_25_ compelling for extreme-condition optoelectronic applications.

**Figure 2.**
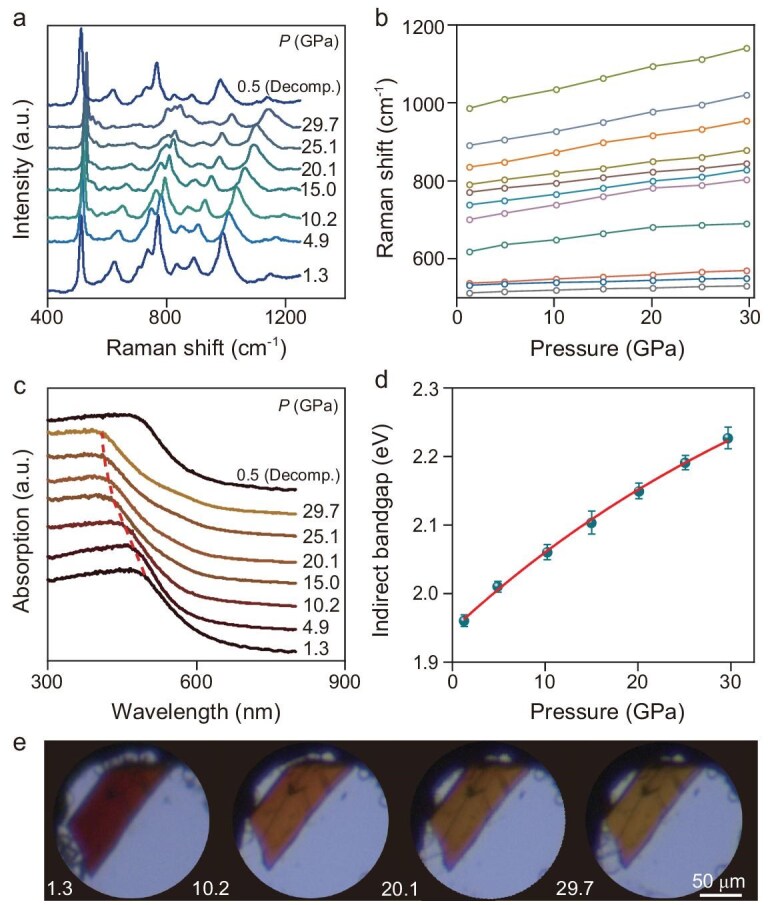
Structure and bandgap evolution of AlCu_1-δ_B_25_ under compression. (a) Raman spectra at representative pressures demonstrating the excellent stability of the tetragonal *P*-4*n*2 phase. (b) Pressure dependence of Raman shifts for all observed vibration modes, confirming lattice stiffening under compression. (c) UV-visible absorption data illustrating the bandgap widening under compression and its reversible behavior upon decompression. (d) Indirect bandgap values as a function of pressure. Experimental uncertainties are from the standard deviation of bandgap values obtained from fitting the absorption pattern with different linear range. (e) Optical microscopy images of single-crystal AlCu_1-δ_B_25_ in a diamond anvil cell at representative pressures. Pressure unit: GPa.

Raman and UV-visible absorption measurements were conducted to study the structural stability and bandgap evolution of AlCu_1-δ_B_25_ under compression. Over the studied pressure range from ambient to 29.7 GPa, all Raman-active modes blue-shift gradually as a function of pressure (Fig. 2a and b), while retaining their spectral profiles with no mode splitting or emergence of additional vibration modes. These observations indicate that the initial tetragonal *P*-4*n*2 phase remains stable throughout the entire studied pressure range, confirming the structural stability of AlCu_1-δ_B_25_ under compression. UV-visible absorption indicates that the absorption edge shows a systematic blue shift toward shorter wavelengths (higher energies) with pressure up to 29.7 GPa (Fig. 2c). Upon decompression to 0.5 GPa, the absorption edge is almost identical to that observed during compression at 1.3 GPa, highlighting the reversibility of the electronic evolutions. Analysis of the absorption data using the Tauc model [41] reveals a monotonic, unsaturated bandgap increase to 2.23 eV when applying pressure of 29.7 GPa (Fig. 2d), indicating an anomalous pressure-induced anti-Wilson effect. This anomaly is further supported by experimental microscopy images (Fig. 2e), where the color of the sample changes from dark red to reddish yellow, indicating the transmission of higher-energy photons and consequent bandgap widening under compression. Bandgap widening typically increases intrinsic resistivity and reduces the dark current of devices. The structural stability and pressure-driven anti-Wilson effect further renders AlCu_1-δ_B_25_ promising for optoelectronic applications under pressure conditions.

**Figure 3.**
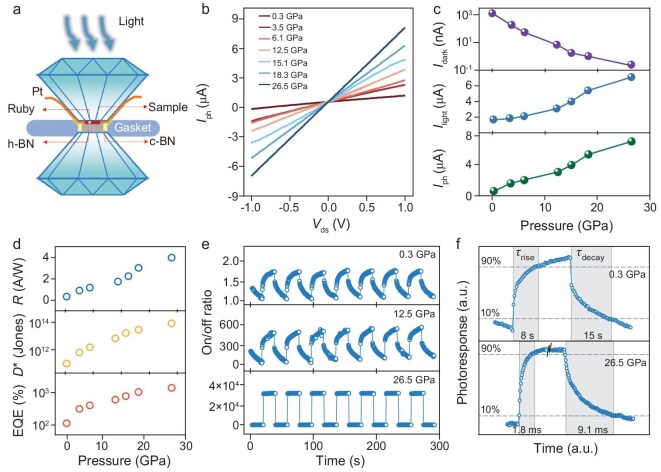
Optoelectronic performance of a AlCu_1-δ_B_25_-based device under an illumination wavelength of 360 nm at representative pressures. (a) Schematic of the high-pressure optoelectronic measurement setup. Hexagonal and cubic boron nitride (h-BN and c-BN) were used as pressure-transmitting media and insulation layer for transport measurements. (b) *I*_ph_ (calculated as *I*_light_−*I*_dark_) as a function of *V*_ds_ within the pressure range of 0.3–26.5 GPa. (c) Pressure-dependent *I*_dark_, *I*_light_, and *I*_ph_ curves. (d) Key photoelectric performance parameters, responsivity (*R*), detectivity (D*), and external quantum efficiency (*EQE*), as a function of pressure. (e) Typical time-resolved photo-response under a power intensity of 0.056 mW/cm^2^ at 0.3, 12.5, and 26.5 GPa. (f) Transient photo-response curves at 0.3 GPa and 26.5 GPa.

We studied the optoelectronic performance of single-crystal AlCu_1-δ_B_25_-based devices under pressure. The main findings are that the overall optoelectronic performances of the AlCu_1-δ_B_25_-based device are significantly enhanced, including increased photocurrent, suppressed dark current, and accelerated photo-response. Figure 3a illustrates the schematic of the *in situ* high-pressure optoelectronic performance measurement setup. Typical *I*_ds_-*V*_ds_ curves of a single-crystal AlCu_1-δ_B_25_-based optoelectronic device, measured in the dark and under illumination with a power intensity of 0.056 W/cm², are shown in Fig. S5. Figure 3b and c presents the evolutions of measured currents under illumination (*I*_light_) and in the dark (*I*_dark_), and photocurrent (*I*_ph_ = *I*_light_—*I*_dark_) as a function of pressure. *I*_light_ rises gradually from ∼1.47 μA at 0.3 GPa to ∼7.22 μA at 26.5 GPa. Interestingly, *I*_dark_ is dramatically suppressed by nearly four-orders of magnitude, from 1.1 × 10^3^ to ∼0.2 nA, causing ∼20-fold enhancement in *I*_ph_ from ∼0.37 to ∼7.22 μA. These advances collectively drive an unprecedented enhancement in the on/off ratio (*I*_ph_/*I*_dark_), exceeding five orders of magnitude. The enhancement of optoelectronic performances with pressure are further characterized by key figures of merit, including responsivity (*R*), detectivity (D*), and external quantum efficiency (*EQE*). As shown in Fig. 3d, the obtained R, D*, and *EQE* values at 26.5 GPa are ∼3.98 A/W, ∼8.59 × 10^13^ Jones, and ∼1.38 × 10^3^%, respectively, which are 1∼2 orders-of-magnitude higher than the corresponding values at 0.3 GPa (∼0.33 A/W, ∼1.01 × 10^11^ Jones, and ∼1.15 × 10^2^%). Typical time-resolved photo-responses at 0.3, 12.5, and 26.5 GPa (Fig. 3e) demonstrate the outstanding repeatability of optoelectronic performance under compression. Notably, the response times (τ_rise_ and τ_decay_) are also significantly improved after pressure modulation (Fig. 3f). The τ_rise_ and τ_decay_ of AlCu_1-δ_B_25_-based optoelectronic devices are ∼1.8 and 9.1 ms at 26.5 GPa, respectively, which are three orders of magnitude faster than those at 0.3 GPa (τ_rise_ = 8 s and τ_decay_ = 15 s). Furthermore, the photocurrent and on/off ratio remain almost unchanged over >1000 switching cycles (Fig. S6), with no observable performance degradation. Additionally, the wavelength-dependent photo-responses at 0.5 and 26.5 GPa (Fig. S7), indicate a broad spectral response, high on/off ratio, and fast response speed at high pressure. These synergistic improvements of the optoelectronic performance under compression suggest the great promise of AlCu_1-δ_B_25_ for harsh-environment devices.

**Figure 4.**
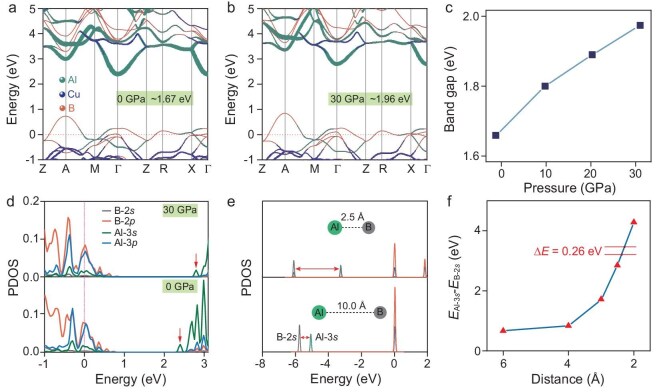
Electronic structure of AlCuB_25_. (a and b) Orbital-resolved band structures at 0 and 30 GPa, respectively. (c) Calculated bandgap values as a function of pressure. (d) Projected density of states (PDOS) at 0 and 30 GPa. (e) Distance-dependent density of states (DOS) of the Al-B two-atom system, showing energy separation between Al-3*s* and B-2*s* states. (f) Energy separation values as a function of interatomic distance between Al and B, based on the two-atom system shown in (e).

Pressure-induced bandgap widening (anti-Wilson effect) has been observed in limited material systems including alkali metals [42–45], alkaline earth metals [46,47], boron carbide [48], and certain perovskites [49]. Reported mechanisms include pressure-induced structural transition [45,46], repulsion of valence electrons by core electrons into lattice interstices [43], and sublattice distortion [49]. The absence of pressure-induced phase transition from Raman data (Fig. 2a and b) rules it out as the likely cause for the anti-Wilson effect. To study the mechanism of the anomalous anti-Wilson effect in compressed AlCu_1-δ_B_25_, first-principles calculations were performed, which confirms the pressure-induced anti-Wilson effect. Prior to systematic calculations, Cu occupancies of 0%, 50%, and 100% were selected to evaluate the influence of Cu proportion on the electronic structure. The calculation results (Fig. S9) reveal that Cu mainly contributes to the valence bands below −1 eV, and has negligible contributions to the valence band maximum (VBM) and conduction band minimum (CBM) which govern electrical transport and optoelectronic properties. Thus, Cu is unlikely to be responsible for the bandgap widening under pressure. For simplicity, fully occupied Cu sites in the 2c/2d Wyckoff positions of the *P*-4*n*2 structure, i.e. AlCuB_25_, were adopted for subsequent calculations. At zero pressure, AlCuB_25_ is a degenerate semiconductor referred to as α-tetragonal boron with an indirect bandgap of ∼1.67 eV, where the VBM and CBM locate at the high-symmetry points of the Brillouin zone, A and Γ, respectively (Fig. 4a). Upon applying pressure up to 30 GPa, the valence bands are almost pressure-insensitive while the Al-dominated CBM bands contract significantly, shifting the CBM to higher energy and thereby widening the bandgap to ∼1.96 eV (Fig. 4b). This pressure-induced bandgap increase (Fig. 4c) (Δ*E* ≈ 0.29 eV from 0 to 30 GPa) is in good agreement with the absorption results (Δ*E* ≈ 0.27 eV).

We found that the pressure-induced bandgap widening correlates to the elevated Al-3*s* conduction bands due to their interactions with the B-2*s* electrons. Projected density of states calculations indicate that the CBM is primarily composed of Al-3*s* states (Fig. 4d and Fig. S10). Considering that Al atoms are mainly bonded with the B atoms in AlCuB_25_, we calculated the electronic structure of two isolated Al and B atoms with different distances, to explore the mechanism of the abnormal bandgap widening. The calculation results show that the Al-3*s* and B-2*s* electrons interact significantly as their interatomic distance decreases, causing an upward shift of the Al-3*s* bands and increasing the energy difference between their dominated states (Fig. 4e and f). Although the hybridization and charge transfer between Al and B atoms in AlCuB_25_ crystals are more complicated, their effect to the Al-3*s* states should be similar. In the case of AlCuB_25_, the distances between an Al atom and its surrounding B atoms decrease from 2.27–2.52 Å at 0 GPa to 2.17–2.41 Å at 30 GPa. Assuming their interactions resemble the isolated Al and B atom model, the energy difference is estimated to be ∆(*E*_Al-3_*_s_*–*E*_B-2_*_s_*) ≈ 0.26 eV (Fig. 4f) from 0 to 30 GPa, which is consistent with the bandgap increase, suggesting its critical role in the anti-Wilson effect of AlCu_1-δ_B_25_ under compression. This pressure-driven orbital separation contributed by two group IIIA elements provides a new perspective for studying the mechanism of the anti-Wilson effect. We also notice that although the B_12_ icosahedra rotate slightly under compression, its contribution to bandgap widening is minimal (Fig. S11), excluding structure distortion as the possible cause for the anti-Wilson effect. Bader charge calculations further support the evolution of the interactions between Al and B, where an obvious electron redistribution occurs between them with increasing pressure (Fig. S12).

## CONCLUSION

We have demonstrated that pressure substantially enhances the optoelectronic performance of boron-rich semiconductor AlCu_1-δ_B_25_ via an anti-Wilson effect. Specifically, AlCu_1-δ_B_25_ displays a monotonic bandgap increase with pressure, which effectively suppresses dark current by three orders of magnitude. This suppression, coupled with a significant photocurrent enhancement, drives a remarkable improvement in the on/off ratio, exceeding 10^5^-fold. Simultaneously, the response time is dramatically reduced by three orders of magnitude with pressure. These synergistic performance optimizations, companied with the excellent structural stability of AlCu_1-δ_B_25_ within a large pressure range, deviate from typical performance degradation and trade-offs observed in materials under compression. The electronic interactions between B-2*s* and Al-3*s* electrons elevate the Al-3*s* conduction bands and drive the anti-Wilson effect in compressed AlCu_1-δ_B_25_. This study not only provides an innovative strategy for performance optimization via the pressure-induced anti-Wilson effect, but also establishes boron-rich semiconductors as compelling candidates for durable electronics operating under extreme conditions, including high-pressure sensors for deep-earth and aerospace environments.

## Supplementary Material

nwag051_Supplemental_File
