# Supplementary material for: Giant enhancement of optoelectronic properties in compressed boron-rich semiconductors
Source: Natl Sci Rev. 2026 Jan 27;13(6):nwag051. doi: 10.1093/nsr/nwag051 (PMC12980326; doi:10.1093/nsr/nwag051)
Supplement: nwag051_Supplemental_File [file nwag051_supplemental_file.pdf]

Supporting Information

Giant Enhancement of Optoelectronic Properties in Compressed Boron-Rich Semiconductors

Ming-Xing Huang,[1,2†] Kun Ye,[3†] Jingyu Hou,[1†] Yufei Gao,[1] Guochun Yang,[2] Lin Wang,[1] Wentao Hu,[1] Bo Xu,[1] Zhongyuan Liu,[1] Xiao-Ji Weng,[1*] Feng Ke,[1*] Xiang-Feng Zhou,[1,2*] and Yongjun Tian[1]

[1]Center for High Pressure Science, State Key Laboratory of Metastable Materials Science and Technology, Yanshan University, Qinhuangdao 066004, China

[2]Hebei Key Laboratory of Microstructural Material Physics, School of Science, Yanshan University, Qinhuangdao 066004, China

[3]School of Electronics and Information Engineering, Institute of Quantum Materials and Devices, State Key Laboratory of Separation Membrane and Membrane Processes, Tiangong University, Tianjin 300387, China

***Corresponding authors.** E-mails: xjweng@ysu.edu.cn; fengke@ysu.edu.cn; xfzhou@ysu.edu.cn

[†]Equally contributed to this work

Methods

Sample synthesis

$AlCu_{1-\delta}B_{25}$ single crystals were synthesized via a high-pressure, high-temperature (HPHT) method. High-purity raw materials, including β-B powders (purchased from Alfa Aesar, 99.999%, particle size <20 μm), Cu powders (99.9%, <80 μm), and Al foil (99.9%), were precisely weighed in a molar ratio of Cu:Al:B = 10:2:1, and loaded into a hexagonal boron nitride (h-BN) crucible. HPHT experiments were performed in a cubic large-volume press with loading pressures of 3-5 GPa and temperatures ranging from 1350 to 1750 K. After maintaining these HTHP conditions for 30 minutes, the samples were quenched to room temperature and ambient pressure. The obtained products were then polished and treated with dilute nitric acid solution to dissolve residual metallic impurities, yielding high-quality, well-faceted single crystals. Pressure calibration was achieved by monitoring the phase transitions of bismuth in the cubic large-volume press, and temperature was measured using a calibrated Pt/Rh thermocouple (Pt-30%Rh vs. Pt-6%Rh), with an estimated temperature uncertainty of ±15 K.

Crystal structure characterizations

Ambient-pressure structure of $AlCu_{1-\delta}B_{25}$ was characterized by powdered XRD using a Rigaku Ultima IV diffractometer (Cu Kα radiation, λ = 0.15406 nm) operating at 40 kV and 40 mA. Morphology and elemental compositions of the synthesized single crystals were analyzed via scanning electron microscopy (Thermo Fisher Verios G4 UC) equipped with energy-dispersive X-ray spectroscopy. Electron-transparent samples for scanning transmission electron microscopy (STEM) analysis were prepared using a focused ion beam milling (Thermo Fisher Helios 5 CX) to ~100 nm in thickness, followed by subsequent cutting to <50 nm using a low-energy Ar^+ polishing (Fischione Model 1040 NanoMill). Prior to the STEM analysis, all samples were subjected to 30-second Ar plasma cleaning (Gatan 695 Plasma Cleaner) to remove surface hydrocarbon contamination. High-angle annular dark-field and Annular-bright-field imaging were collected using an aberration-corrected STEM system (Thermo Fisher Themis Z) at an accelerating voltage of 300 kV.

Electrical and mechanical property measurements

Temperature-dependent resistivity and Hall effect measurements were conducted in a Quantum Design Physical Property Measurement System (PPMS-9) across the temperature range of 2–

300 K. A ±5 T magnetic field was applied for Hall effect measurements. Electrical contacts were established on a plate-shaped single-crystal sample using four gold wires (~15 μm in diameter) bonded with conductive silver pastes. Vickers microhardness (H_v) testing was performed on single crystals using a KB 5 BVZ tester with a square pyramidal diamond indenter. A series of loads (0.049 – 0.686 N) were applied with consistent 20 s loading and dwell periods. Hardness values were calculated according to: $H_v = 1854.4F/L^2$, where F represents the applied load (N) and L is the mean diagonal length (μm). The asymptotic hardness value was determined from ≥5 measurements per load condition.

High-pressure absorption and Raman measurements

High-pressure optical absorption and Raman measurements were performed using a symmetric-type diamond anvil cell, equipped with a pair of type-IIa anvils (500 μm culet in diameter). Sample chamber was prepared using the following procedure: pre-indenting a T301 stainless steel gasket (~250 μm initial thick), drilling a hole with 400 μm in diameter in the center of the indentation area using a home-built laser-drilling equipment, encapsulating an insulating layer (mixture of epoxy and cubic boron nitride with 1:10 in weight ratio) on the indentation area, and drilling an additional 250 μm-diameter hole on it, serving as the sample chamber. Pressure was calibrated using ruby fluorescence. Silicone oils were employed as the pressure-transmitting media to provide quasi-hydrostatic pressure conditions. UV-Visible absorption spectra were acquired in transmission mode using an Ocean Optics QE65000 spectrometer. The absorption spectra were obtained by subtracting the backgrounds from silicone oil and diamond anvils from raw transmission data. Raman spectra were collected using a Horiba HR Evolution confocal micro-Raman system equipped with a 532 nm excitation laser. The spectrometer was calibrated before each experiments using the Raman shift of a single-crystal silicon reference at 520.7 cm^{-1}.

High-pressure optoelectronic property measurements

High-pressure photoelectric property measurements were conducted on the same diamond anvil cell used for absorption or Raman. Fine h-BN powders with particle size of ~20 nm were used as pressure-transmitting medium to ensure a quasi-hydrostatic pressure condition. Electrical contacts were established by directly pressing Pt electrodes onto the surface of $AlCu_{1-\delta}B_{25}$ single crystal. Photoelectric response data for illumination of various wavelengths (360,

457, and 532 nm) were acquired using a Keithley 2634B Source. The spot size of the 360, 457, and 532 nm lasers is approximately 1.2 mm in diameter. Signals of the temporal response of the phototransistors were obtained using an oscilloscope equipment with an amplifier. The incident light was modulated with an optical chopper (SR540, Stanford Research System). Responsivity (R), detectivity (D^*), and external quantum efficiency (EQE) were calculated based on the following formulas: $R = I_{ph}/PS$, $D^* = RS^{1/2}/(2qI_{dark})^{1/2}$, $EQE = hcR/q\lambda$, where P, S, q, h, c, and λ is power intensity, effective area, element charge, Planck's constant, light velocity, and laser wavelength, respectively. I_{ds}-V_{ds} curves were collected prior to all optoelectronic measurements to evaluate the contacts between the metal electrodes and $AlCu_{1-\delta}B_{25}$ single crystal (Fig. 3b). Temperature-dependent I_{ds}-V_{ds} measurements from 160 to 300 K were also performed to quantitatively determine the Schottky barrier height (Φ_B) (Fig. S8a).

DFT calculations

Electronic structure calculations were performed using the PWmat package based on a plane-wave basis set [1,2]. Optimized norm-conserving Vanderbilt pseudopotentials [3,4] and the generalized gradient approximation (GGA) with the Perdew-Burke-Ernzerhof (PBE) exchange-correlation functional [5] were initially applied for structural relaxation of both lattice parameters and atomic positions. A plane-wave cutoff energy of 60 Ry and uniform k-point grids with a resolution of $2\pi \times 0.03$ Å^{-1} were set to ensure convergence, with forces on each atom below 0.001 eV/Å^{-1}. For more accurate bandgap determinations, self-consistent field and band structure were further calculated using the hybrid Heyd-Scuseria-Enrzerhof (HSE06) functional [6-8]. Pressure was applied by setting a target stress in variable-cell structural optimization, based on the method developed in previous studies [9,10], which has been implemented in the PWmat package. All other calculations were performed based on the corresponding optimized structure at the target pressure.

Table S1. Structural details of the synthesized copper-doped boron-rich semiconductor $AlCu_{1-\delta}B_{25}$.

Atom	Site	*x*	*y*	*z*	Occupancy
Al	4e	0.00000	0.00000	0.04870	0.5
Al	4e	0.00000	0.00000	-0.04870	0.5
Cu1	2c	0.00000	0.50000	0.25000	0.760
Cu2	8d	0.50000	0.00000	0.25000	0.028
B1	8i	0.32590	0.08320	0.40500	1.000
B2	8i	0.23690	0.08090	0.08500	1.000
B3	8i	0.13040	0.12320	0.38030	1.000
B4	8i	0.09010	0.23030	0.08560	1.000
B5	8i	0.09370	0.31400	0.41280	1.000
B6	8i	0.24850	0.24040	0.58480	1.000
B7	2b	0.00000	0.00000	0.50000	1.000

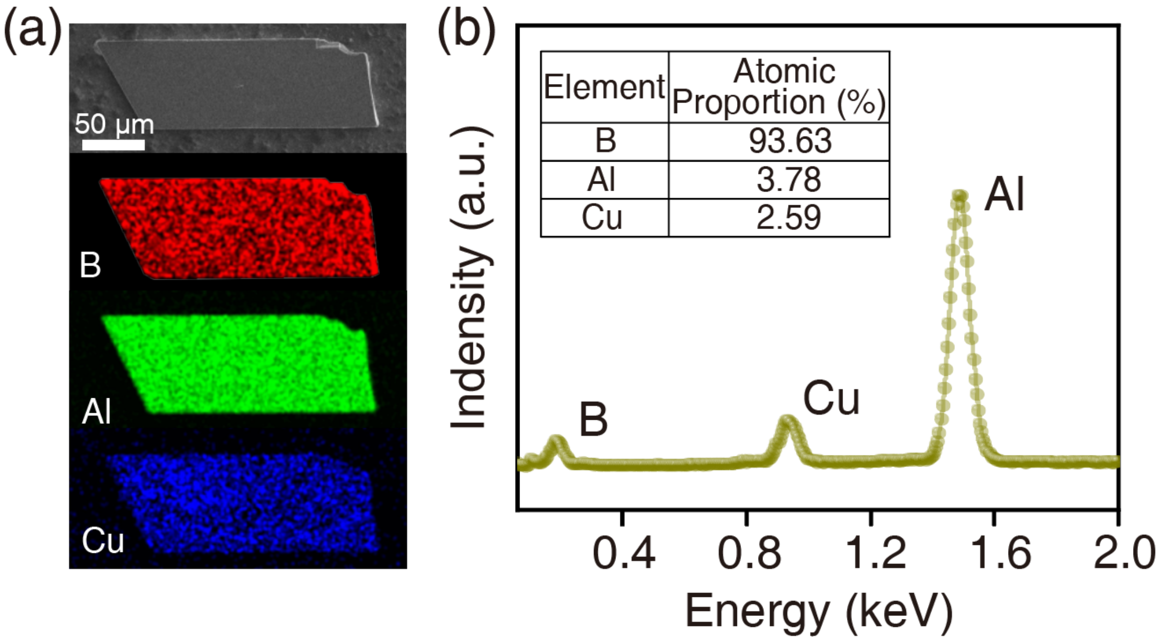

Figure S1. Scanning electron microscopy (SEM) characterization of the synthesized $AlCu_{1-\delta}B_{25}$ single crystal. (a) SEM image and element distribution analysis results. (b) Energy-dispersive X-ray spectroscopy. Inset: obtained average proportions of B, Al and Cu elements.

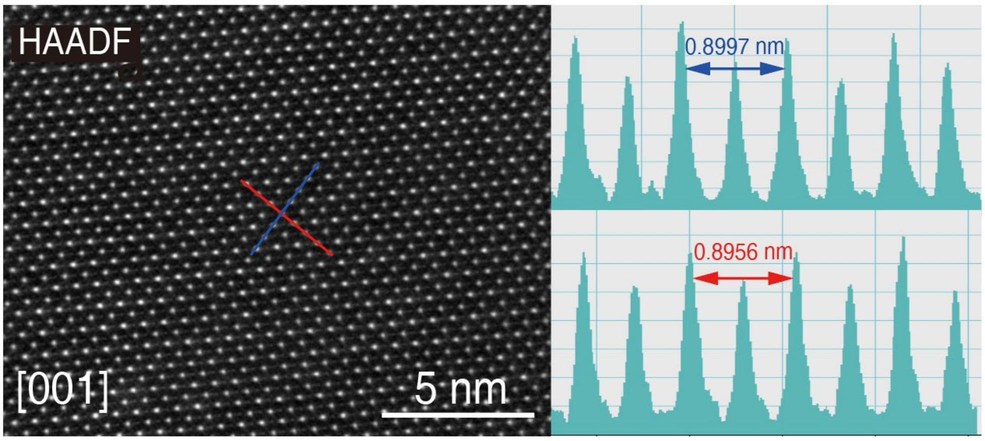

Figure S2. High-angle annular dark-field images of $AlCu_{1-\delta}B_{25}$ single crystals along the [001] axis.

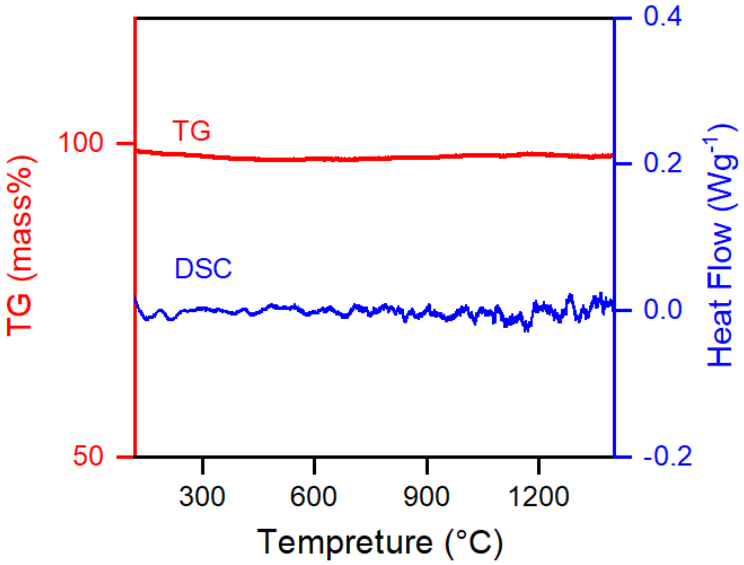

Figure S3. Differential scanning calorimetry (DSC, blue curve) and thermal gravimetric analysis (TG, red curve) of $AlCu_{1-\delta}B_{25}$ under argon protection.

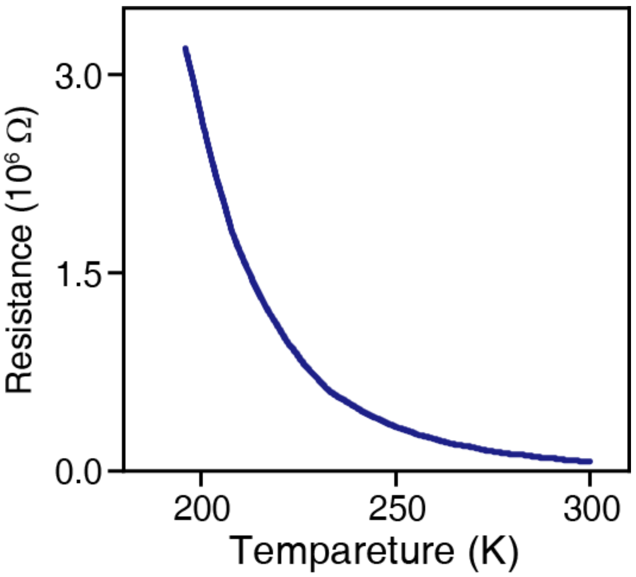

Figure S4. Temperature dependent resistance of single-crystal $AlCu_{1-\delta}B_{25}$ measured at ambient pressure.

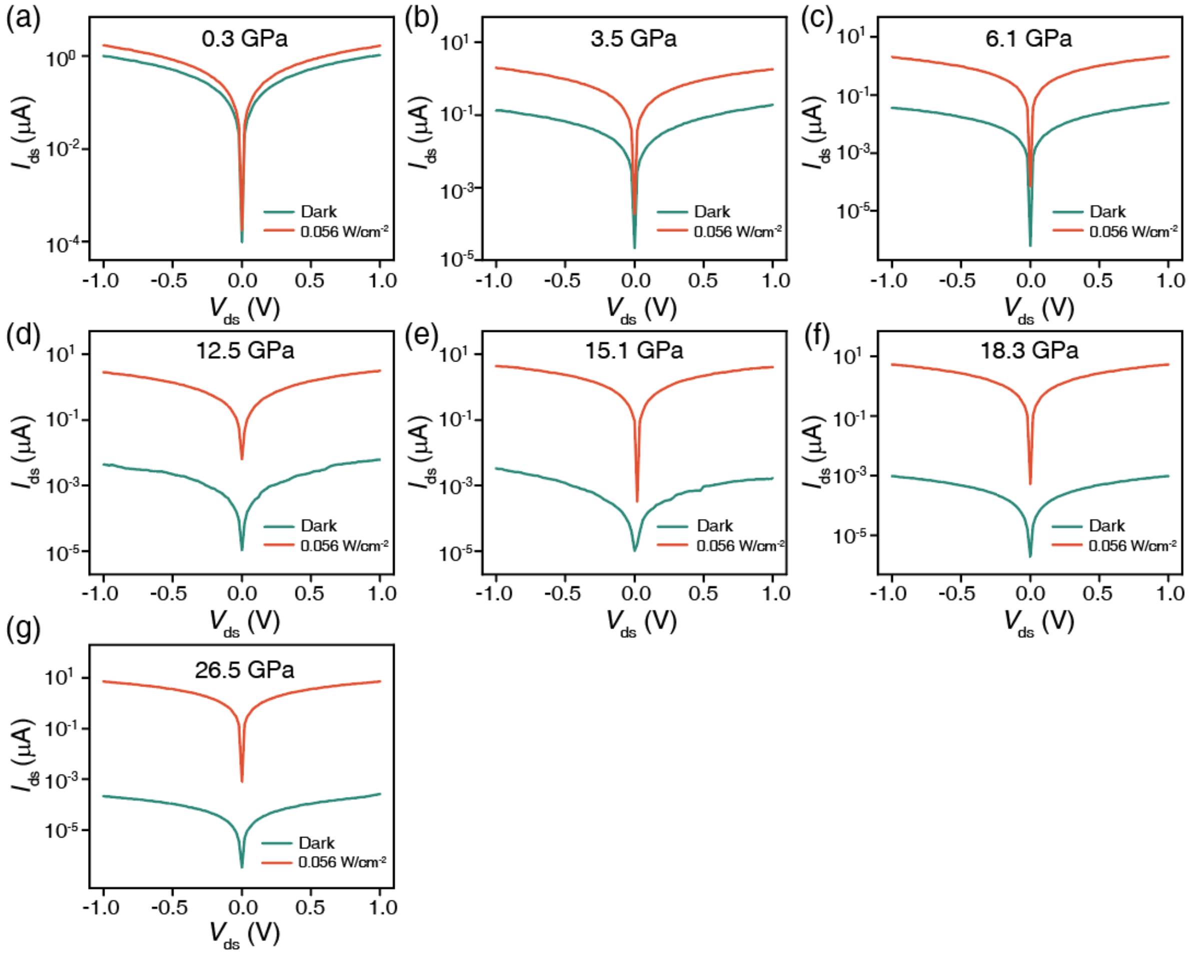

Figure S5. Typical drain-source current vs voltage (I_{ds}-V_{ds}) curves of a single-crystal $AlCu_{1-\delta}B_{25}$-based optoelectronic device in the dark and under illumination with power intensity of 0.056 W/cm^2 under compression. I_{ds} is in log scale.

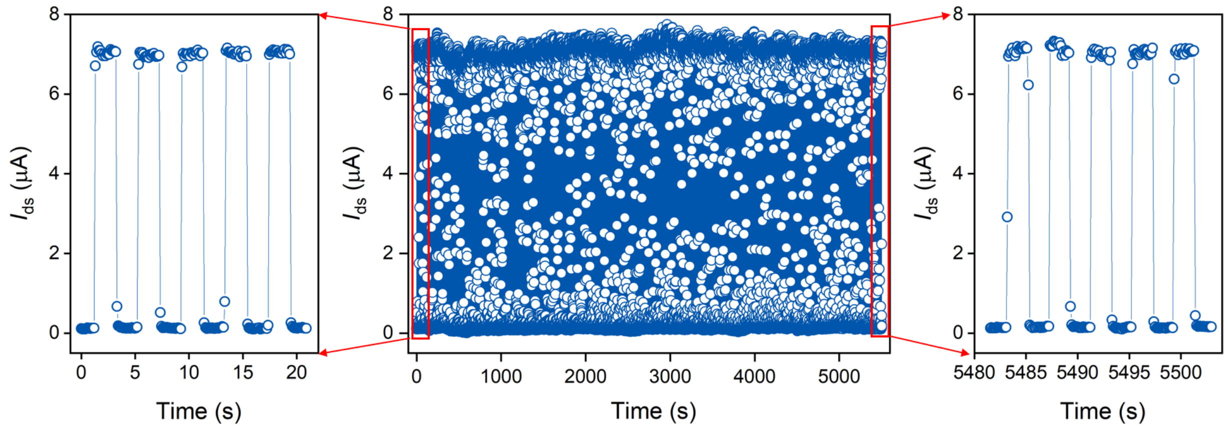

Figure S6. Cycling stability test of $AlCu_{1-\delta}B_{25}$-based optoelectronic device over >1000 switching cycles.

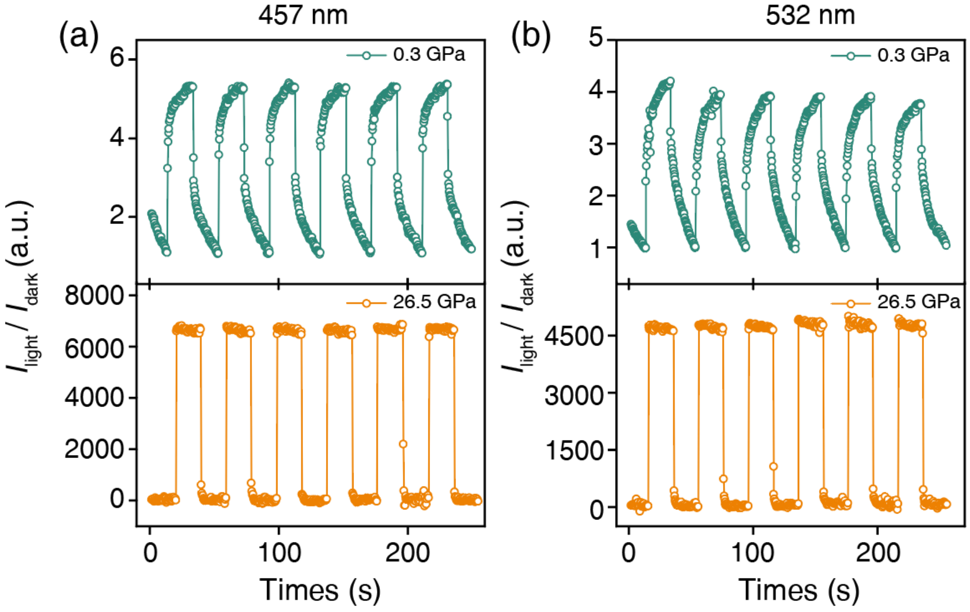

Figure S7. Time-resolved photo-responses at 0.5 GPa and 26.5 GPa under illumination wavelengths of 457 nm (a) 532 nm (b).

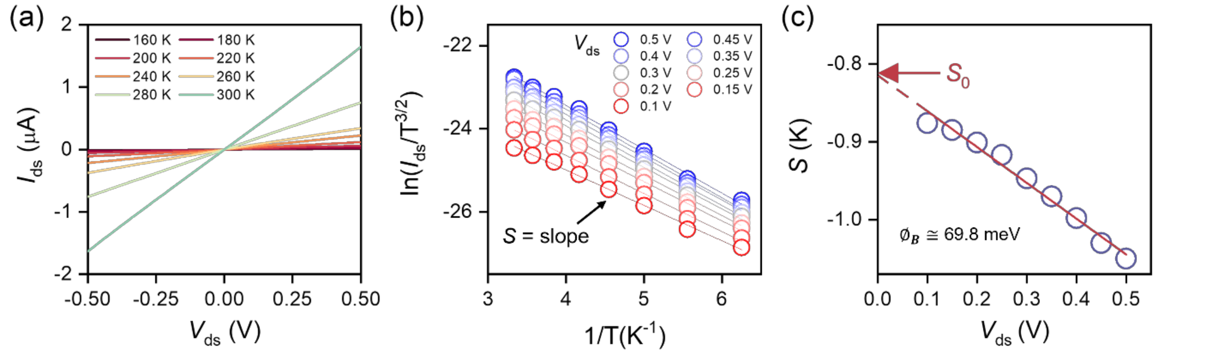

Figure S8. (a) Temperature-dependent I_{ds}-V_{ds} characteristics measured from 160 to 300 K. The linear and symmetric I_{ds}-V_{ds} curves provide direct evidence of ohmic contacts between the electrodes and the $AlCu_{1-\delta}B_{25}$ single crystal. (b) Corresponding Arrhenius plots of $\ln(I_{ds}/T^{3/2})$ versus $1/T$ at selected bias voltages. (c) Extracted slope S as a function of V_{ds}. At each drain bias, the current was analyzed using Arrhenius plots to extract the slope S. The voltage dependence of S was then examined and linearly extrapolated to $V_{ds} = 0$ (Fig. S8b, c), yielding the zero-bias slope S_0. Using the relation $S_0 = \frac{-q\Phi_B}{1000k_B}$, we calculated a barrier height $\Phi_B \approx 69.8$ meV. This value is lower than the 100 meV threshold for high-quality ohmic contacts established in the literature [11,12]. Such a low barrier enables that thermionic emission is minimally impeded. Consequently, carrier injection is highly efficient, photo-generated carriers can be effectively collected, and the reported optoelectronic performance is reliable.

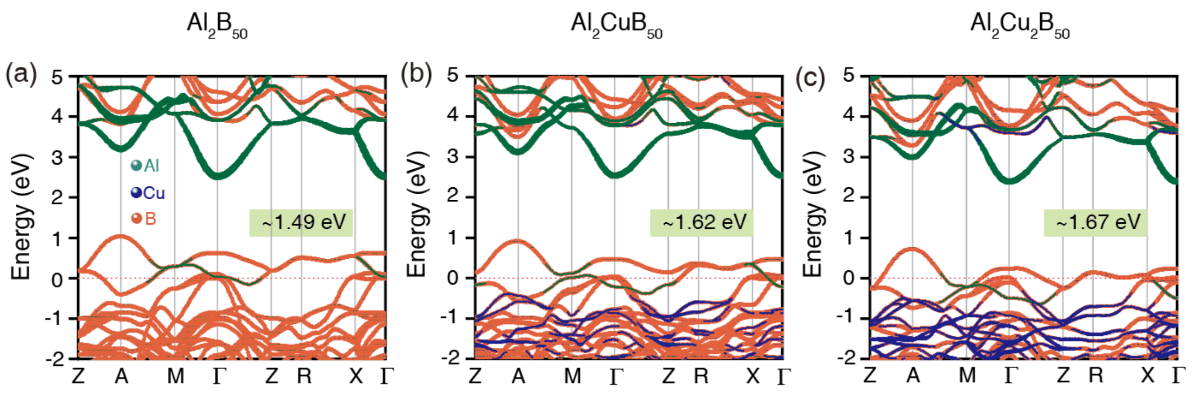

Figure S9. Band structures of Al_2B_{50} (a), Al_2CuB_{50} (b), and $Al_2Cu_2B_{50}$ (c) at 0 GPa, respectively. Contributions from Al, Cu, and B elements are marked in different colors.

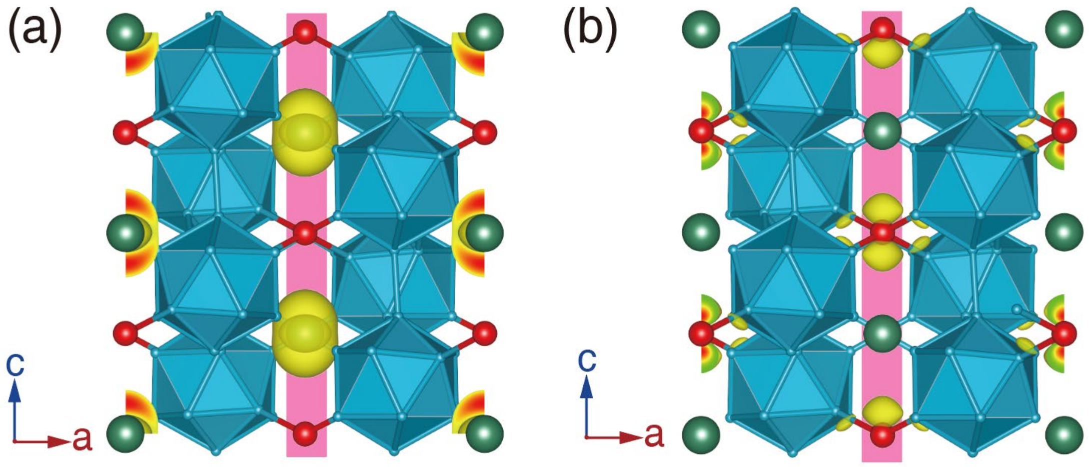

Figure S10. Partial charge densities corresponding to the CBM (a) and VBM (b). The nearly spherical bubbles around Al atoms and dumbbell-shaped bubbles around interstitial B atoms confirm that the Al-3*s* and B-2*p* states contribute most to CBM and VBM, respectively. Pink regions highlight the conductive channels in $AlCu_{1-\delta}B_{25}$.

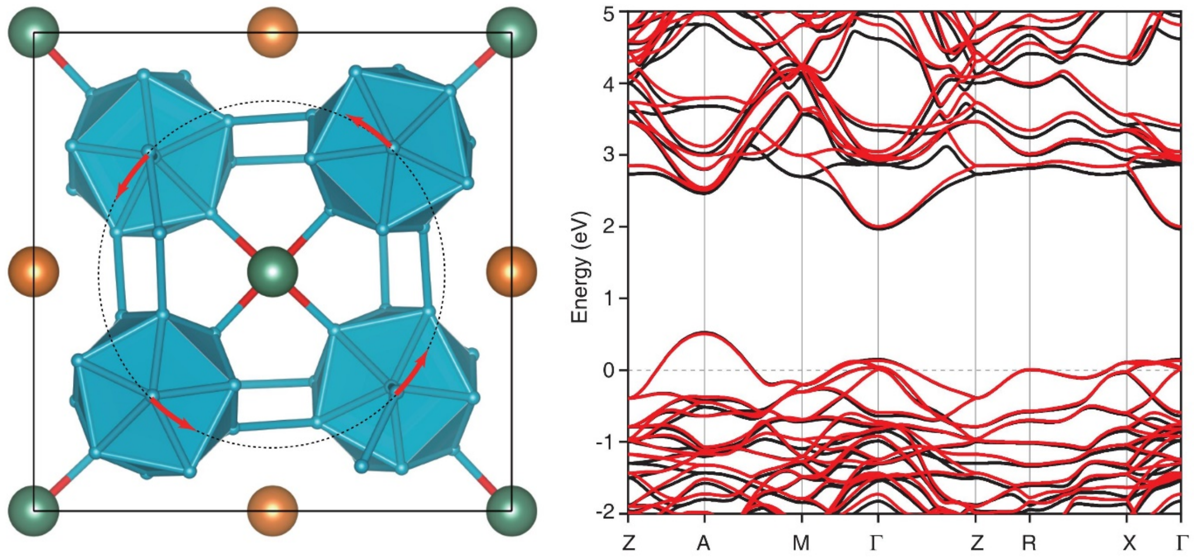

Figure S11. (a) Fully optimized structure of $AlCuB_{25}$ at 30 GPa. B_{12} icosahedra rotate slightly (marked by the red arrows) compared to the structure freezing the atomic coordination to the zero-pressure values. (b) Electronic structure of $AlCuB_{25}$ at 30 GPa using different structure models. Black bands: the fully optimized structure at 30 GPa. Red bands: structure model using the 30 GPa lattices but freezing the atomic coordinates at their 0 GPa values. The similarity between the two band structures at 30 GPa indicates that slight rotation of the B_{12} icosahedra has minimal contributions to the observed bandgap widening under pressure.

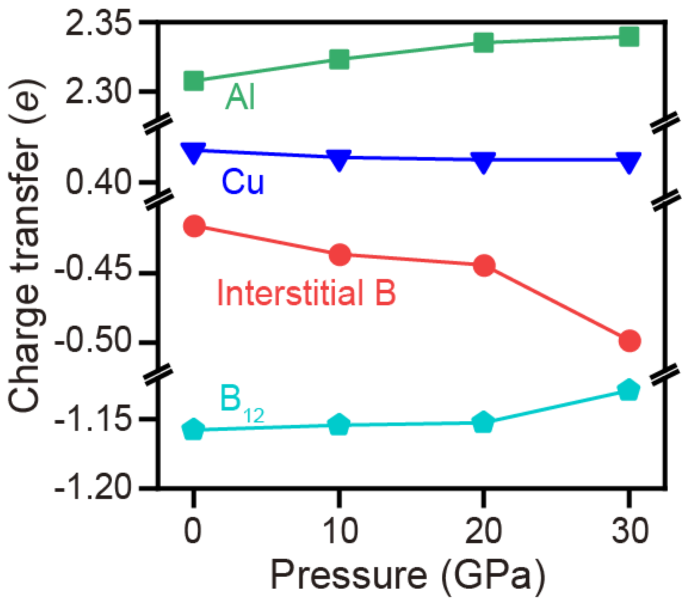

Figure S12. Calculated Bader charge as a function of pressure for Al, Cu, B_{12} and interstitial B in $AlCuB_{25}$. The results indicate that Al and Cu atoms are positively charged and act as electron donors, whereas the B_{12} icosahedra and interstitial B sites accept the electrons. Notably, the Bader charge of Al atom is approximately +2.3, characteristic of a trivalent cation and indicative of strong ionic interactions with the boride framework. With increasing pressure, the charge on Cu atoms remains relatively constant, while significant electron redistribution occurs between Al and B, which plays a sizable contribution to the enhanced interactions between Al-3s and B-2s states, as well as the observed anti-Wilson effect.

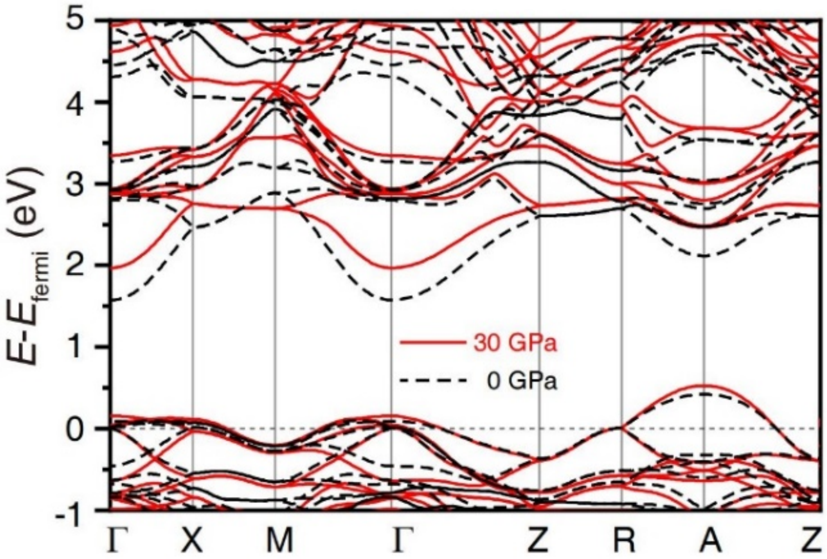

Figure S13. Band structures of $AlCuB_{25}$ calculated using the VASP software with the PBE functional. The calculated bandgap increases from ~1.15 eV at 0 GPa to ~1.44 eV at 30 GPa, corresponding to a pressure-induced bandgap widening of 0.29 eV. While the PBE functional systematically underestimates the absolute bandgap value compared to the HSE06 hybrid functional, the calculated bandgap widening values from these two methods are in excellent agreement (Fig. 4c).

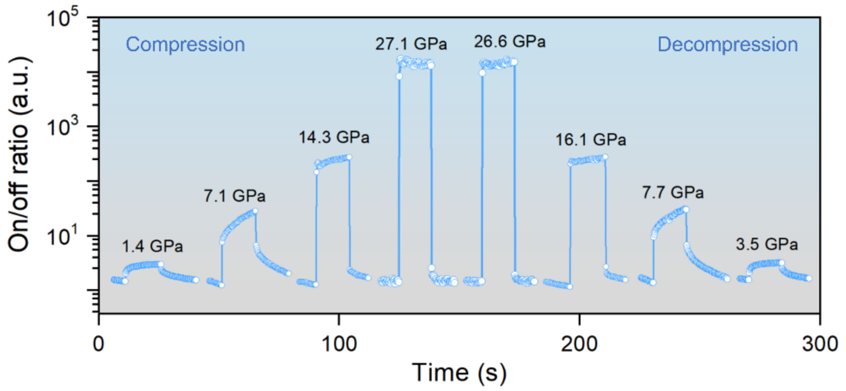

Figure S14. Optoelectronic performance of $AlCu_{1-\delta}B_{25}$-based optoelectronic device throughout a compression-decompression cycle. The device performance remains stable and fully reversible throughout the cycle.

References

1. Jia W, Cao Z, Wang L *et al.* The analysis of a plane wave pseudopotential density functional theory code on a GPU machine. *Comput Phys Commun* 2013; **184**: 9-18.
2. Jia W, Fu J, Cao Z *et al.* Fast plane wave density functional theory molecular dynamics calculations on multi-GPU machines. *J Comput Phys* 2013; **251**: 102-15.
3. Hamann DR. Optimized norm-conserving Vanderbilt pseudopotentials. *Phys Rev B* 2013; **88**: 085117.
4. Schlipf M, Gygi F. Optimization algorithm for the generation of ONCV pseudopotentials. *Comput Phys Commun* 2015; **196**: 36-44.
5. Perdew JP, Burke K, Ernzerhof M. Generalized gradient approximation made simple. *Phys Rev Lett* 1996; **77**: 3865-8.
6. Heyd J, Scuseria GE, Ernzerhof M. Hybrid functionals based on a screened Coulomb potential. *J Chem Phys* 2003; **118**: 8207-15.
7. Heyd J, Scuseria GE. Efficient hybrid density functional calculations in solids: Assessment of the Heyd–Scuseria–Ernzerhof screened Coulomb hybrid functional. *J Chem Phys* 2004; **121**: 1187-92.
8. Paier J, Marsman M, Hummer K *et al.* Screened hybrid density functionals applied to solids. *J Chem Phys* 2006; **124**: 154709.
9. Nielsen OH, Martin RM. First-principles calculation of stress. *Phys Rev Lett* 1983; **50**: 697-700.
10. Nielsen OH, Martin RM. Quantum-mechanical theory of stress and force. *Phys Rev B* 1985; **32**: 3780-91.
11. Das S, Chen H-Y, Penumatcha AV *et al.* High performance multilayer MoS_2 transistors with scandium contacts. *Nano Lett* 2013; **13**: 100-5.
12. Lee S, Tang A, Aloni S *et al.* Statistical study on the Schottky barrier reduction of tunneling contacts to CVD synthesized MoS_2. *Nano Lett* 2016; **16**: 276-81.
